# Expanding the supply of human leukocytes for research by harvesting cell concentrates from Trima Accel tubing sets

**DOI:** 10.1038/s41598-026-64945-3

**Published:** 2026-08-05

**Authors:** Katharina Kronenberg, Gunther Glehr, Viola Haehnel, Frauke Dormann, Irene Pamler, Giulia Preverin, Fabiola Arella, Michael Kapinsky, Paloma Riquelme, Robert Offner, Hans J. Schlitt, Philipp Beckhove, Edward K. Geissler, Ralph Burkhardt, James A. Hutchinson, Andreas Brosig

**Affiliations:** 1 Department of Surgery, University Hospital Regensburg, Regensburg, Germany; 2 Institute of Clinical Chemistry and Laboratory Medicine, Transfusion Medicine, University Hospital Regensburg, Regensburg, Germany; 3 Beckman Coulter Life Sciences GmbH, Krefeld, Germany; 4 Division of Interventional Immunology, Leibniz Institute for Immunotherapy (LIT), Regensburg, Germany

**Keywords:** Biological techniques, Immunology

## Abstract

Leukoreduction system (LRS) chambers from the Trima Accel apheresis platform are widely used as a convenient source of human peripheral blood mononuclear cells (PBMCs) for immunological research. However, increasing demand has prompted the search for alternative sources. Here, we investigated whether residual cell concentrates recovered from Trima Accel tubing sets, typically discarded after platelet apheresis, could serve as a substitute for concentrates obtained from LRS chambers. Paired samples from 20 healthy platelet donors were analyzed for recovered volume, cellular composition and yield, leukocyte subset distribution, and functional responsiveness. Tubing sets contained significantly greater volumes (23.04 ± 14.1 ml vs. 8.14 ± 0.97 ml) but lower leukocyte concentrations (35.19 ± 16.99 × 10³/µl vs. 147.60 ± 59.63 × 10³/µl) than LRS chambers. Absolute leukocyte numbers were slightly higher in LRS chambers. However, cell frequencies in tubing sets showed a minor enrichment of monocytes and a modest lymphocyte depletion. Detailed flow cytometry characterization further revealed comparable distributions of major leukocyte populations and T cell subsets, including naïve and memory compartments, without biologically meaningful differences in activation or differentiation markers. Importantly, PBMCs from both sources exhibited comparable functional responses following phytohemagglutinin (PHA) stimulation, including similar changes in activation marker expression and cytokine production. Short-term storage of tubing set samples for 24 h at 4 °C did not compromise cell viability. Overall, tubing sets represent a functionally equivalent and practical alternative leukocyte source for research applications.

**Supplementary Information:**

The online version contains supplementary material available at 10.1038/s41598-026-64945-3.

## Introduction

Human peripheral blood mononuclear cells (PBMC) collected as a by-product of platelet donation are widely used in immunological research. In particular, Leukoreduction System (LRS) chambers from the Trima Accel^®^ apheresis platform are a well-validated, convenient and high-yield source of fresh leukocytes for experimentation^1–4^. LRS chambers are intended to remove leukocytes from platelet products to enhance transfusion safety^5,6^. Cell concentrates that remain trapped in so-called “cones” at the end of apheresis can be easily recovered for PBMC isolation^7,8^. Owing to an increasing emphasis on human research and the shift away from animal experiments, our center has experienced a significant rise in demand for PBMCs. This demand has surpassed current supply, necessitating the identification of alternative sources.

The Trima Accel^®^ apheresis system employs a sterile, single-use disposable tubing kit (Trima Accel^®^ LRS^®^ Platelet, Plasma Set, Catalog No. 82300) with the LRS chamber integrated into that kit. A considerable volume of blood components remains within the tubing set after completion of the apheresis procedure and is typically discarded. Recent studies have characterized the cellular content of LRS chambers and the remaining disposable tubing system after platelet apheresis, with particular focus on the effects of plasma rinseback on residual leukocyte populations^9^.

**Fig. 1:**
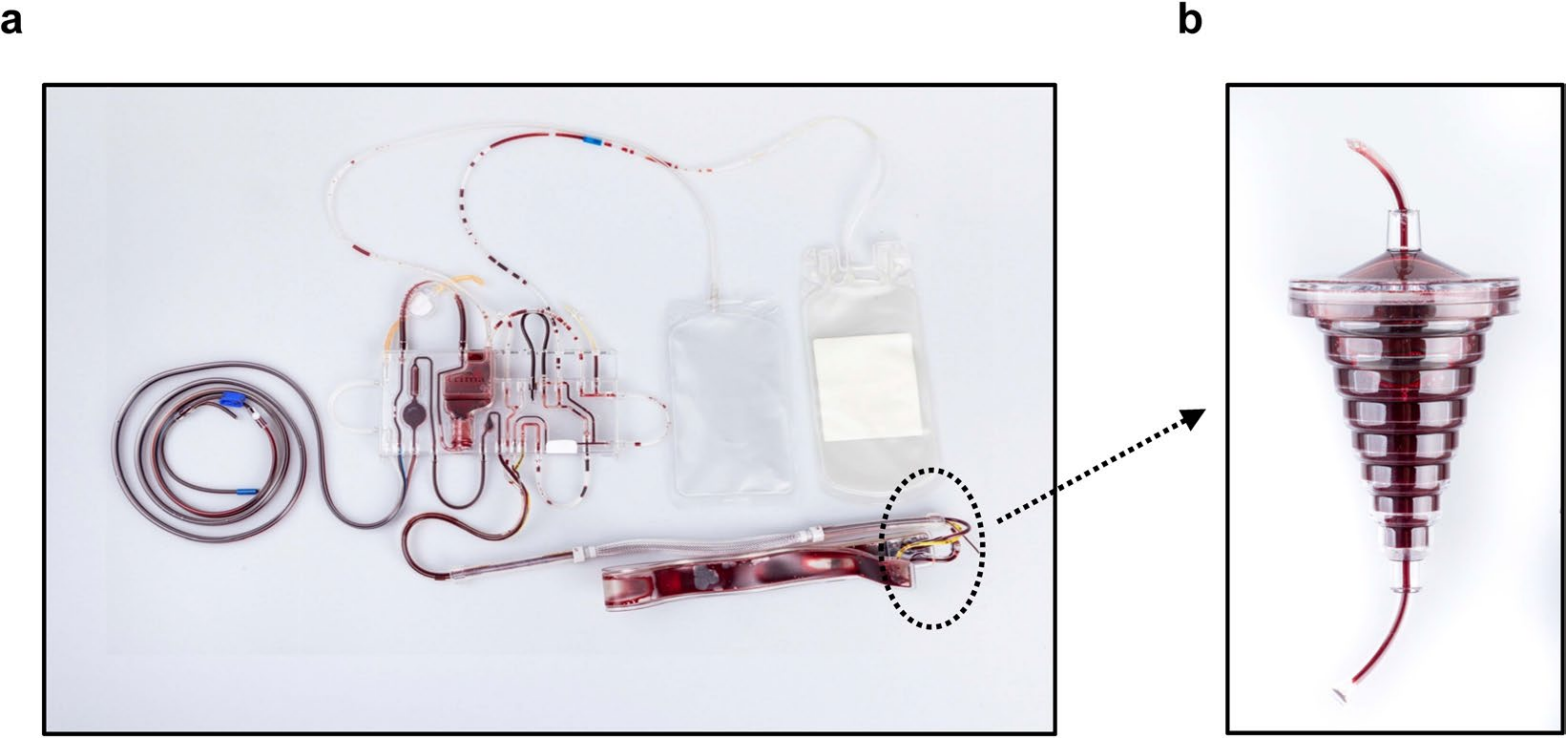
Disposable components of the Trima Accel^®^ apheresis device. (**a**) Photograph of a used tubing set removed from a Trima Accel^®^ apheresis device after platelet donation. (**b**) Photograph of a used Leukoreduction System (LRS) chamber taken from a Trima Accel^®^ apheresis device after platelet donation.

The objective of the present study was to evaluate the quality of concentrates recovered from the tubing set (kit without LRS chamber) following platelet apheresis without rinseback (Fig. 1a) and to compare them with leukocyte products obtained from LRS chambers (Fig. 1b). In particular, we analyzed differences in cellular composition, yield of various leukocytes subsets, and functional responses in vitro. In addition, the quality of leucocytes from tubing sets was evaluated after storage.

## Results

### Comparison of leukocyte concentrates from LRS chambers and tubing sets

**Fig. 2:**
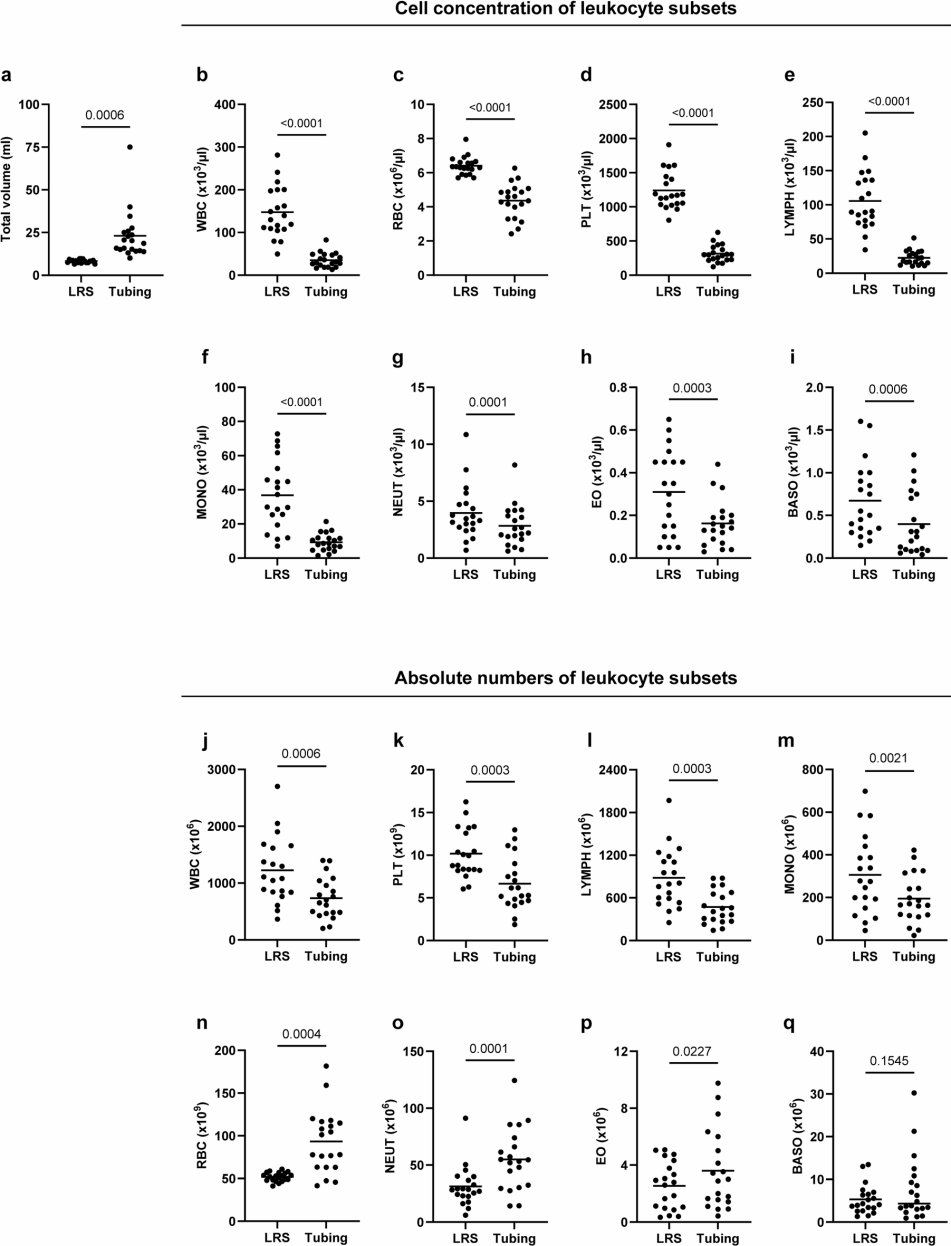
Comparison of leukocyte concentrates from LRS chambers and tubing sets. LRS chambers contained lower volumes of cell concentrate at higher cell density than tubing sets (*n* = 20 donors; paired t-test with Benjamini-Hochberg correction). (**a**) Volume of cell concentrates from paired LRS chambers and tubing sets. Leukocyte subset concentrations from paired LRS chambers and tubing sets were determined using a hematology cell counter: (**b**) white blood cells (WBC); (**c**) red blood cells (RBC); (**d**) platelets (PLT); (**e**) lymphocytes (LYMPH); (**f**) monocytes (MONO); (**g**) neutrophils (NEUT); (**h**) eosinophils (EO); (**i**) basophils (BASO). Absolute cell numbers recovered from paired LRS chambers and tubing sets were calculated from recovered volumes and subset frequencies: (**j**) WBC; (**k**) PLT; (**l**) LYMPH; (**m**) MONO; (**n**) RBC; (**o**) NEUT; (**p**) EO; (**q**) BASO.

Paired samples of leukocyte concentrates were collected from LRS chambers or tubing sets from 20 healthy platelet donors (Table S1). The total volume of each sample was measured and its cellular composition was assessed using an automated cell counter. The recovered volume from the tubing set was significantly greater than from LRS chambers (23.04 ± 14.1 ml vs. 8.14 ± 0.97 ml, *n* = 20, *p* = 0.0006; Fig. 2a). Concentrations of all leukocyte subsets were significantly lower in cell concentrates from tubing sets compared to LRS chambers (Fig. 2b–i). However, owing to the larger volume of concentrates recovered from tubing sets, overall numbers of leukocytes (WBC), platelets (PLT), lymphocytes (LYMPH) and monocytes (MONO) contained in tubing sets was only marginally lower compared to LRS chambers (Fig. 2j–m). Tubing set samples contained higher absolute numbers of red blood cells (RBC), neutrophils (NEUT) and eosinophils (EO) (Fig. 2n–p). No differences were observed with regard to basophils (BASO) (Fig. 2q).

**Fig. 3:**
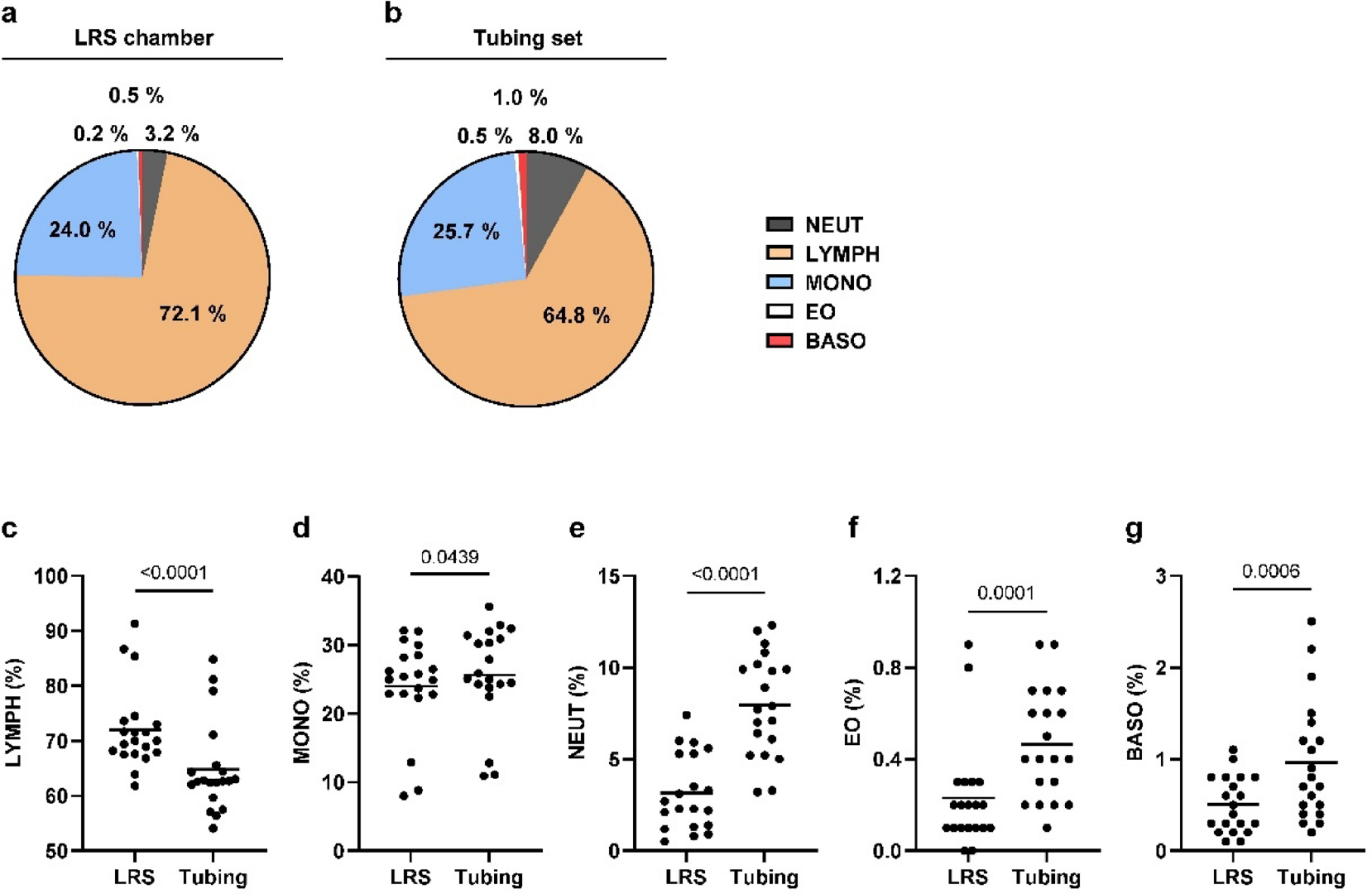
Comparison of leukocyte subset frequencies from LRS chambers and tubing sets. Mean proportions of leukocyte subsets in paired samples of cell concentrates from (**a**) LRS chambers and (**b**) tubing sets (*n* = 20 donors). Relative abundances of (**c**) lymphocytes (LYMPH), (**d**) monocytes (MONO), (**e**) neutrophils (NEUT), (**f**) eosinophils (EO), and (**g**) basophils (BASO) from paired samples of cell concentrates from LRS chambers and tubing sets were determined using a hematology cell counter (*n* = 20 donors; paired t-test with Benjamini-Hochberg correction).

The relative numbers of leukocyte subsets were similar in LRS chambers (Fig. 3a) and tubing sets (Fig. 3b). The proportion of lymphocytes was slightly decreased in tubing sets (64.79 ± 8.20% vs. 72.07 ± 7.51%, *n* = 20, *p* < 0.0001) (Fig. 3c). By contrast, MONO, NEUT, EO and BASO were somewhat enriched in tubing set samples (Fig. 3d-g). Hence, LRS chambers yielded slightly more leukocytes than tubing sets, but the overall cellular composition recovered from both was very similar.

### Flow cytometry analysis of leukocyte concentrates from LRS chambers and tubing sets

**Fig. 4:**
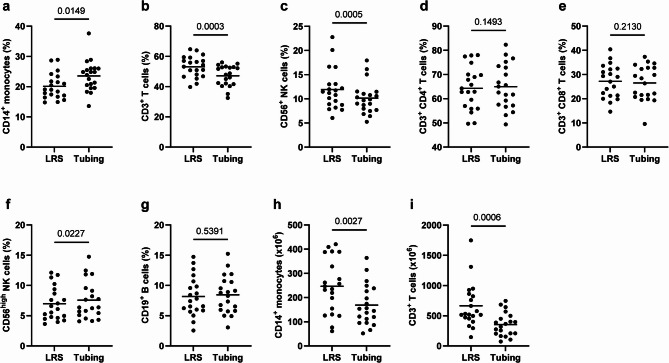
Flow cytometry analysis of leukocyte concentrates from LRS chambers and tubing sets. Samples of cell concentrates from paired LRS chambers and tubing sets were analyzed by flow cytometry analysis using the DURAClone IM Phenotyping Basic Tube. Leukocyte subset frequencies were assessed by manual gating for (**a**) CD14^+^ monocytes gated on CD45^+^ leukocytes, (**b**) CD3^+^ T cells gated on CD45^+^ leukocytes, (**c**) CD56^+^ NK cells gated on CD45^+^ leukocytes, (**d**) CD4^+^ T cells gated on CD3^+^ T cells, (**e**) CD8^+^ T cells gated on CD3^+^ T cells, (**f**) CD56^high^ NK cells gated on CD56^+^ NK cells, and (**g**) CD19^+^ B cells gated on CD45^+^ leukocytes. Absolute numbers of total recovered (**h**) CD14^+^ monocytes and (**i**) CD3^+^ T cells were calculated (*n* = 20 donors; paired t-test with Benjamini-Hochberg correction).

Paired cell concentrates from LRS chambers and tubing sets were further characterized by flow cytometry analysis and leukocyte subset frequencies were extracted by manual gating (Figure S1). Flow cytometry analysis confirmed higher proportion of CD14^+^ monocytes in tubing set samples than in LRS chambers (Fig. 4a). A decreased proportion of CD3⁺ T cells (Fig. 4b) and CD56⁺ NK cells (Fig. 4c) was observed in concentrates from tubing sets. No significant differences were detected between LRS and tubing set groups in the frequencies of CD4⁺ T cells (Fig. 4d) and CD8⁺ T cells (Fig. 4e), CD56^high^ NK cells (Fig. 4f) or CD19⁺ B cells (Fig. 4g). Total CD14^+^ monocyte recovery from tubing sets (168.5 ± 84.7 × 10^6^ cells) was significantly lower than from LRS chambers (246.3 ± 113.3 × 10^6^ cells) (Fig. 4h). Likewise, fewer total CD3^+^ T cells were recovered from tubing sets (356.0 ± 195.3 × 10^6^ cells) compared to LRS chambers (665.0 ± 369.9 × 10^6^ for CD3^+^ T cells) (Fig. 4i).

Detailed flow cytometric analyses of T cell subsets did not reveal any biologically relevant differences between LRS chambers and tubing sets (Figure S2). Notably, the distribution of CD4⁺ and CD8⁺ memory T cell subsets was comparable between both leukocyte concentrates, including effector memory (T_EM_), effector memory RA⁺ (T_EMRA_), central memory (T_CM_) and naïve T cells (Figure S3a–h). Likewise, the expression profiles of T cell activation and differentiation markers, including CD27, CD28, CD57, and PD-1 did not differ between samples from LRS chambers and tubing sets (Figure S3i–p). Hence, only minor differences in leukocyte subset distributions were found between LRS chambers and tubing sets.

### Enrichment of monocytes in tubing sets compared to LRS chambers

**Fig. 5:**
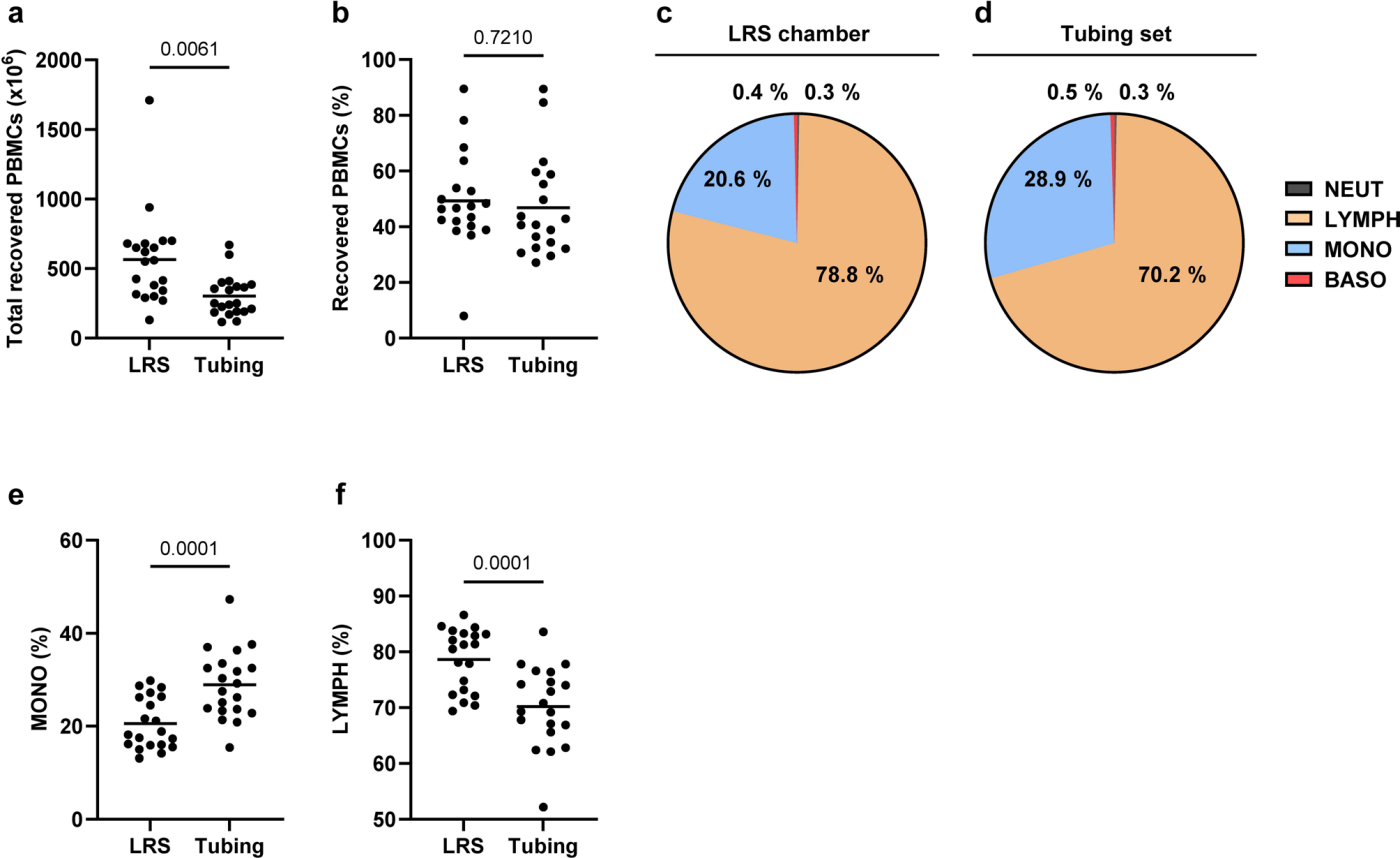
Enrichment of monocytes in PBMC isolated from tubing sets compared to LRS chambers. (**a**) Total numbers of PBMCs recovered from LRS chambers and tubing sets after ficoll density gradient centrifugation and washing (*n* = 20 donors; paired t-test with Benjamini-Hochberg correction). (**b**) Efficiency of ficoll density gradient separation shown as proportion of recovered PBMCs from paired LRS and tubing set cell concentrates (*n* = 19 donors; paired t-test with Benjamini-Hochberg correction). Mean proportions of leukocyte subsets in paired samples of PBMCs isolated from (**c**) LRS chambers and (**d**) tubing sets (*n* = 20 donors). Relative abundances of (**e**) monocytes (MONO), and (**f**) lymphocytes (LYMPH) in PBMCs isolated from paired samples of cell concentrates from LRS chambers and tubing sets were determined using a hematology cell counter (*n* = 20 donors; paired t-test with Benjamini-Hochberg correction).

Our research group studies monocyte-derived macrophages, so we were particularly interested to confirm that monocytes obtained from tubing sets were not inferior to those from LRS chambers in quality or quantity^10^. PBMC were isolated from concentrates by density gradient centrifugation and then analyzed using an automated cell counter. Isolation of PBMCs from tubing set samples resulted in significantly lower total yields compared with PBMCs obtained from LRS chambers (302 × 10^6^ ± 148 × 10^6^ cells vs. 565 × 10^6^ ± 336 × 10^6^ cells, *n* = 20, *p* = 0.0061) (Fig. 5a). Using the percentage of recovered PBMCs as a measure of ficoll density separation efficiency, no significant differences were observed between cell concentrates from LRS chamber and tubing set (49.26 ± 17.29% vs. 46.86 ± 17.80%, *n* = 19, *p* = 0.7210) (Fig. 5b). PBMCs isolated from LRS chambers (Fig. 5c) compared to tubing sets (Fig. 5d) contained a lower proportion of MONO (20.59 ± 5.54% vs. 28.92 ± 7.40%, *n* = 20, *p* = 0.0001) (Fig. 5e). In contrast, the relative number of LYMPH was reduced in tubing sets (70.21 ± 7.21% vs. 78.66 ± 5.58%, *n* = 20, *p* = 0.0001) (Fig. 5f).

### Analysis of PHA-stimulated PBMC from LRS chambers and tubing sets

PBMCs isolated from LRS chamber or tubing set samples were cultured for 22 h in the absence or presence of 5 µg/ml phytohemagglutinin (PHA) and subsequently stained for flow cytometry using the DURAClone IM T Cell Subsets panel. Frequencies of T cell subsets were quantified by manual gating (Figure S2). Across all evaluated populations, PBMCs derived from LRS chamber and tubing set exhibited comparable responses to PHA stimulation, with no detectable differences between the two sources (Figure S4).

**Fig. 6:**
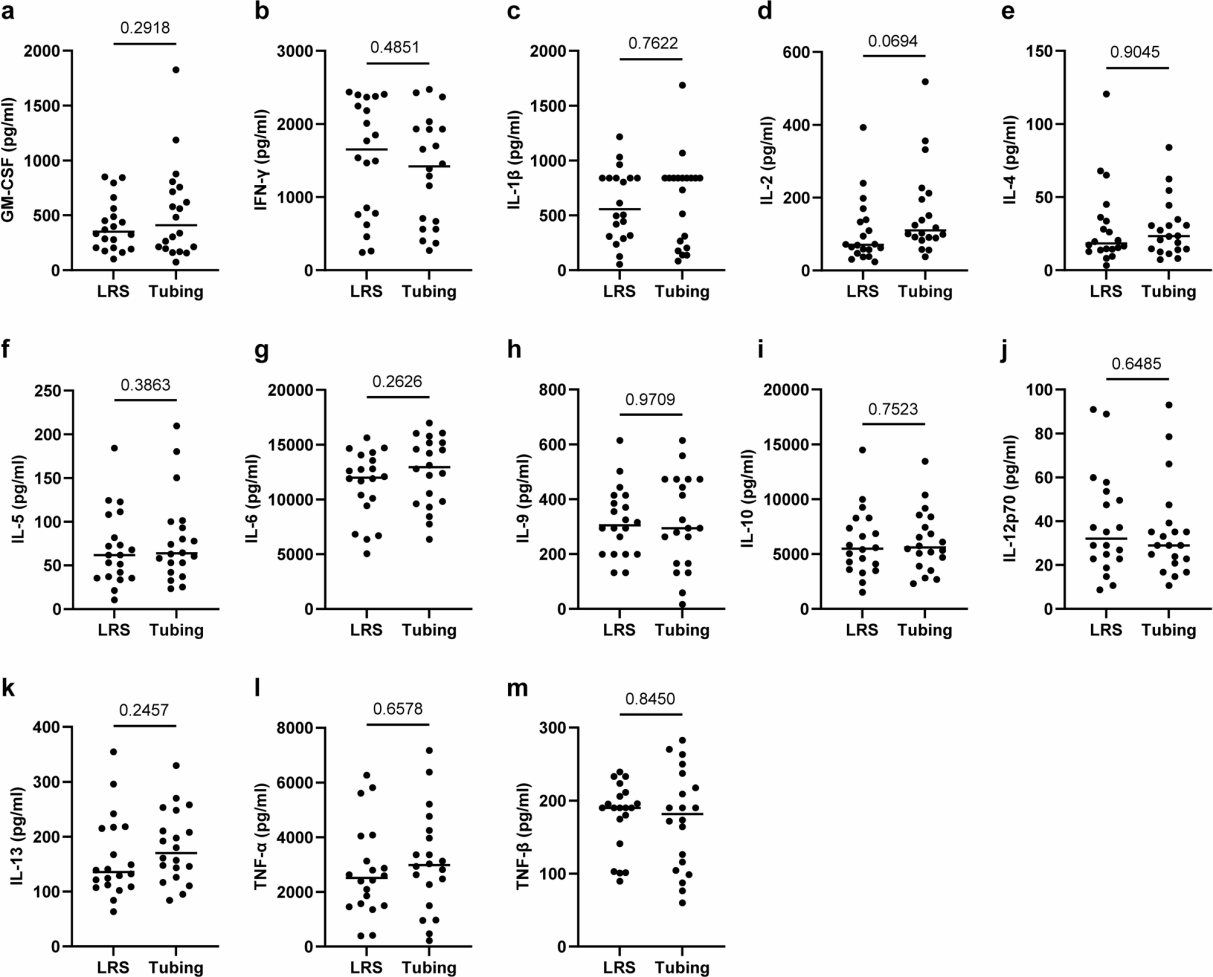
Analysis of cytokine release by PHA-stimulated PBMC from LRS chambers and tubing sets. PBMCs from paired LRS chambers and tubing sets were cultured for 22 h in the presence of 5 µg/ml phytohaemagglutinin (PHA). Cytokine secretion profile was analyzed by Luminex Assay: (**a**) GM-CSF; (**b**) IFN-γ; (**c**) IL-1β; (**d**) IL-2; (**e**) IL-4; (**f**) IL-5; (**g**) IL-6; (**h**) IL-9; (**i**) IL-10; (**j**) IL-12p70; (**k**) IL-13; (**l**) TNF-α; (**m**) TNF-β (*n* = 20 donors; paired t-test with Benjamini-Hochberg correction).

Supernatants from PHA-stimulated (Fig. 6) and control PBMC cultures (Figure S5) were analyzed using a Luminex-based TH1/TH2 cytokine panel that incorporated GM-CSF, IFN-γ, IL-1β, IL-2, IL-4, IL-5, IL-6, IL-9, IL-10, IL-12p70, IL-13, TNF-α and TNF-β. Across all 13 cytokines assessed, there was no measurable difference in the capacity of PBMCs obtained from LRS chambers and tubing sets to react to strong polyclonal T cell stimulus (Fig. 6a-m).

### Viability assessment of tubing set samples during storage

As platelet collections are performed throughout the day, overnight storage would conveniently allow processing of cells on the next day if we could verify that the quality of leuocytes remains stable. It was recently reported that LRS chamber cell concentrates remain stable and viable after overnight storage^11,12^. To assess the viability of tubing set cell concentrates during overnight storage, cells were stained with 7-aminoactinomycin D (7-AAD) and analyzed by flow cytometry (Figure S6). Paired samples from 12 individual donors (Table S2) were collected immediately after donation (day 0) and after 24 h of storage (day 1). Plasma bags containing the tubing set cell suspension were stored either at 4 °C without agitation or at 22 °C under continuous agitation. No significant reduction in cell viability was observed after 24 h, irrespective of the storage conditions (Figure S7). However, samples stored at 22 °C with agitation exhibited tendencies towards lower leucocyte and monocyte viability.

## Discussion

The present study aimed to compare cellular products obtained from the Trima Accel^®^ apheresis system using LRS chamber and tubing set with respect to cellular composition, leukocyte yield, stability and functional responsiveness. Cell concentrates from tubing set were more diluted than those obtained from LRS chambers so that, despite a higher recovered volume from tubing sets, 1.7-fold more leukocytes were recovered from LRS chambers. This finding contrasts with reports from other studies that demonstrated no significant differences in WBC cell counts between LRS and tubing set samples^9^, potentially owing to methodological differences. Recovered volumes from tubing set samples exhibited greater variability than those obtained from LRS chambers. This wider distribution is likely attributable to the recovery procedure. Ficoll separation efficiency was comparable between both sample sources, indicating that sample dilution did not influence the isolation process.

Recovery of major leukocyte populations from LRS chambers and tubing sets was surprisingly consistent with previously reported findings^9^, despite significant differences in the volume of cell suspension (Table S3). In contrast to earlier reports, we observed a bias towards LRS chambers containing more lymphocytes (especially T cells and NK cells) than tubing sets. Vice versa, we also found a relative enrichment of monocytes and granulocytes (NEUT, EO and BASO) in tubing sets compared to LRS chambers. These differences presumably reflect a separation based upon cell size or density owing to different fluid flow in LRS chambers and tubing sets, but the precise mechanism is presently unknown. We did not identify changes in leukocytes recovered from LRS chambers compared to tubing sets, but we cannot rule-out that turbulence affects the quality of leukocytes trapped in LRS chambers.

PBMCs isolated from LRS chamber and tubing set samples were stimulated with phytohemagglutinin (PHA), a potent polyclonal activator. Stimulated PBMCs from LRS chambers and tubing sets demonstrated comparable cytokine responses. This broad measure of the capacity of T cells to respond to strong mitogen stimulation indicates that leucocytes from LRS chambers and tubing sets behave similarly, but do not prove that T cells from both sources are functionally identical in every respect.

Storage of tubing set samples at 4 °C for up to 24 h without agitation did not result in a detectable reduction in cell viability. In contrast, storage at 22 °C under continuous agitation was associated with a trend toward decreased viability of leukocytes and monocytes. These findings suggest that refrigerated storage better preserves cellular integrity and viability during short-term storage.

Due to larger sample volumes the handling of tubing set samples generally increases processing time and workload, as samples may need to be divided across multiple Ficoll gradients. This can lead to increased reagent consumption and variability between preparations.

In the present study, no plasma rinseback was performed at the end of the apheresis procedure. However, plasma rinseback is routinely incorporated into the workflow at many centers to mitigate loss of lymphocytes in frequent platelet donors^13,14^. Previous work from our group demonstrated that inclusion of a rinseback reduced the number of cells retained within LRS chambers^1^, suggesting that a rinseback-dependent reduction in cell numbers may also occur in tubing set products, as recently reported by Kelly et al.^9^.

To mitigate shortages of leukocyte sources for research purposes, end-users should carefully consider the number and type of cells required for their intended applications. Given the substantially higher overall leukocyte yields obtained from LRS chambers compared with tubing sets, allocation strategies could, in principle, be adapted according to the expected abundance of the target cell population. In this context, LRS chambers may be preferentially allocated to studies requiring relatively scarce cell populations, such as monocytes, whereas tubing sets may provide sufficient material for applications focusing on more abundant populations, including conventional T cells. Of course, the distinction between scarce and abundant cell types is highly application-dependent. The number of cells required for a particular downstream assay varies greatly, ranging from relatively small numbers for phenotypic analyses to substantially larger numbers for functional assays or multi-omics applications. Furthermore, donor-to-donor variability, differences in cell recovery during processing, and the purity requirements of individual experiments may substantially influence the suitability of a given leukocyte source. Consequently, the allocation of LRS chamber versus tubing set should be guided by the specific experimental objectives and cell number requirements. A potential advantage of such an allocation strategy is the more efficient utilization of available blood donation by-products and improved access to rare cell populations for research, thereby maximizing the scientific value derived from these materials.

## Methods

### Apheresis procedure

Apheresis from healthy platelet donors were performed by the Department of Transfusion Medicine at University Hospital Regensburg using the Trima Accel^®^ automated blood collection system (Terumo BCT, Inc., Lakewood, USA) with software version 7 and 100% plasma (without additive solution) for platelet collection. Apheresis was run with an ACDA ratio of 1:10 and 3.5–4.5 L processed blood volume and without rinseback. Following completion of the apheresis procedure, the LRS chamber was sterilely welded while the tubing system (Tubing Set 82300 Trima Accel^®^ LRS Platelet, Plasma Set) remained installed within the device, thereby preserving the sterility of the closed system. The tubing set was subsequently removed from the device and rotated by 180°, positioning the attached empty plasma bag inferior to the remaining tubing system. The set was then suspended vertically from a hook to facilitate gravity-assisted handling during the subsequent processing steps. To recover the residual cell concentrate retained within the tubing system, the air trap was manually compressed while the tubing and attached components were gently agitated. This combination of controlled pressure and agitation enabled the remaining cell suspension to be displaced through the tubing and collected in the attached empty plasma bag. The transfer was continued until no further cell concentrate passed through the tubing. Following completion of the transfer, the plasma bag containing the recovered cell concentrate was sterilely welded and separated from the tubing system for subsequent processing.

This study was authorized by the local ethics committee of the University of Regensburg (approval 22-2780-101). All experiments were performed in accordance with the ethical standards of the institutional research committee and the Declaration of Helsinki. Written informed consent for participation and the use of biological samples for research purposes was obtained from all 32 participants. Samples were collected from JUL-2025 until MAY-2026.

### Automated analysis of cell concentrates

Leukocyte concentrates were recovered from LRS chambers or plasma bag and total volume was determined using a serological pipette. LRS cell concentrates were diluted with PBS (1:5), whereas tubing set cell suspension were used undiluted. Their cellular composition was analyzed using an automated cell counter (XN-550, Sysmex, Norderstedt, Germany) returning values for white blood cells (WBC), red blood cells (RBC), platelets (PLT), lymphocytes (LYMPH), monocytes (MONO), neutrophils (NEUT), eosinophils (EO) and basophils (BASO).

### Clinical flow cytometry

Cell concentrates were stained for flow cytometry using the DURAClone IM Phenotyping Basic Tube (B53309) and the DURAClone IM T Cell Subsets Tube (B53328, both Beckman Coulter, Krefeld, Germany) according to standard operating procedure^15^. Data were recorded with a Navios^™^ cytometer running Cytometry List Mode Data Acquisition and Analysis Software version 1.3 (Beckman Coulter). Analyses were performed using Kaluza software version 2.2 (Beckman Coulter) as described elsewhere^16^. One donor was excluded from the memory T cell subset analysis owing to a CD45RA mutant phenotype.

### Viability assessment

Following platelet apheresis, tubing set cell concentrates were transferred into the empty plasma bag. For viability testing, 1 mL aliquots were aseptically collected using a Product Sampling Set (HPF 0612, CELL-MAX GmbH, Munich, Germany) that had been sterile-welded to the plasma bag. Tubing set cell concentrates were stored for 24 h either at 4 °C without agitation or at 22 °C under continuous agitation at 60 cycles/min.

Cell viability was assessed by flow cytometry using 7-aminoactinomycin D (7-AAD; IM3614, Beckman Coulter, Krefeld, Germany) in combination with monoclonal antibodies against CD3 (A94680), CD14 (A99020), and CD45 (B36294; all Beckman Coulter). Heat-inactivated cells were included as positive controls for non-viable cells. Erythrocytes were lysed using VersaLyse Lysing Solution (A09777, Beckman Coulter) according to the manufacturer’s instructions. Subsequently, cells were incubated with Fc receptor blocking reagent (130-059-901, Miltenyi Biotec, Bergisch Gladbach, Germany), followed by staining with the respective surface antibodies. After washing, cells were stained with 7-AAD and immediately acquired on a Navios™ flow cytometer using Cytometry List Mode Data Acquisition and Analysis Software version 1.3 (Beckman Coulter). Data analysis was performed using Kaluza software version 2.2 (Beckman Coulter).

### Cell culture experiments

The LRS chamber and the plasma bag from the tubing set were each transferred to separate 50 mL conical tubes and allowed to drain under gravity. PBMCs were enriched by Ficoll density gradient centrifugation, as previously described^10,17^. In brief, diluted blood was layered over Ficoll-Paque and centrifuged without brake. The PBMC layer at the plasma-Ficoll interface was collected, washed with PBS, and pelleted for downstream analysis. Due to the larger sample volumes, cell concentrates derived from tubing sets required processing with two Ficoll gradients, whereas cell products obtained from LRS chambers were processed using a single Ficoll preparation. Isolated PBMCs were seeded at a density of 3 × 10⁶ cells/ml in TexMACS medium (130-097-196, Miltenyi Biotec, Bergisch Gladbach, Germany) supplemented with Antibiotic-Antimycotic (1524006, Thermo Fisher Scientific, Dreieich, Germany). Cells were cultured for 22 h at 37 °C in a humidified atmosphere containing 5% CO₂, either in the absence or presence of phytohemagglutinin (PHA; L1668, Sigma-Aldrich, Taufkirchen, Germany) at a final concentration of 5 µg/ml. After 22 h of incubation, culture supernatants were collected, and cells were harvested for subsequent flow cytometric analysis.

### Luminex assay

Cytokine concentrations in the supernatants of cultured PBMCs were quantified using the fixed human Th1/Th2 High Performance Luminex Assay (LKTM008B, Bio-Techne, Abingdon, United Kingdom), in accordance with the manufacturer’s instructions. Briefly, samples and standards were prepared as specified in the protocol and incubated with fluorescently labeled capture beads, followed by detection antibody and streptavidin–phycoerythrin incubation. Plates were washed between incubation steps according to the recommended procedure. Data were recorded using the Bio-Plex MAGPIX Multiplex Reader running xPONENT^®^ data acquisition and analysis software.

### Statistics and visualisation

Data visualisation and statistical analyses were performed using GraphPad Prism version 10.3.1 (GraphPad Software, Inc., Boston, USA). Statistics were calculated using the paired t-test with normality assumption and the Benjamini-Hochberg correction.

## Supplementary Information

Below is the link to the electronic supplementary material.

Supplementary Material 1

## Data Availability

Data are available from the corresponding author upon reasonable request.
