# Supplementary material for: Expanding the supply of human leukocytes for research by harvesting cell concentrates from Trima Accel tubing sets
Source: Sci Rep. 2026 Aug 5;16:24195. doi: 10.1038/s41598-026-64945-3 (PMC13443108; doi:10.1038/s41598-026-64945-3)
Supplement: Supplementary file 1 [file 41598_2026_64945_MOESM1_ESM.pdf]

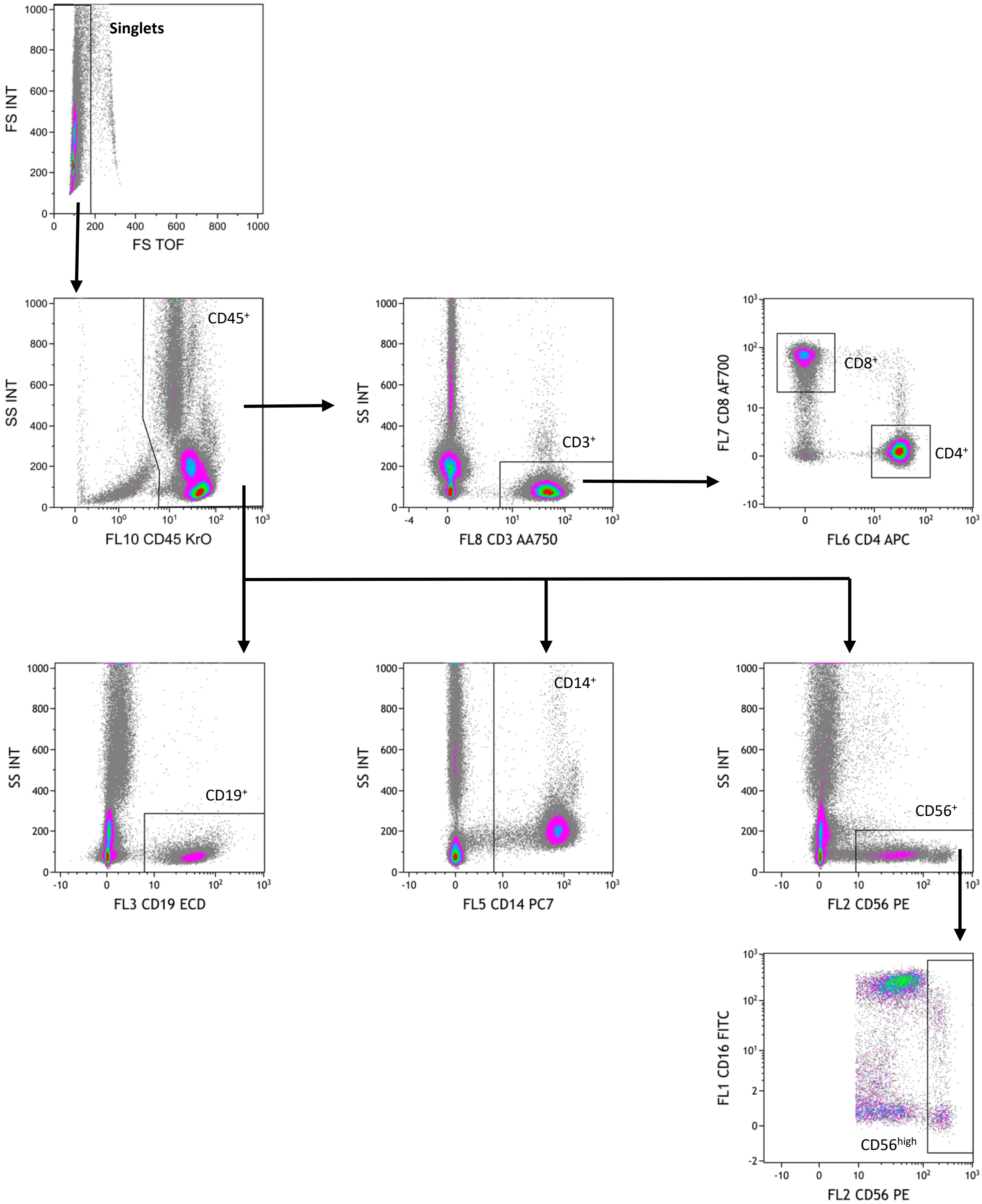

Supplementary Figure 1. *Gating strategy of the DURAClone IM Phenotyping Basic Tube.* Cell concentrates were stained using the dried-down DURAClone IM Phenotyping Basic Tube (Beckman Coulter) and analyzed by flow cytometry. Sequential gating included selection of singlets, identification of $CD45^+$ leukocytes, and gating of $CD3^+$ T cells, with subsequent discrimination of $CD4^+$ and $CD8^+$ T cell subsets. Within the $CD45^+$ population, $CD19^+$ B cells, $CD14^+$ monocytes, and $CD56^+$ NK cells were identified, and $CD56^{high}$ NK cells were gated within the $CD56^+$ compartment. A representative gating from a single sample is shown.

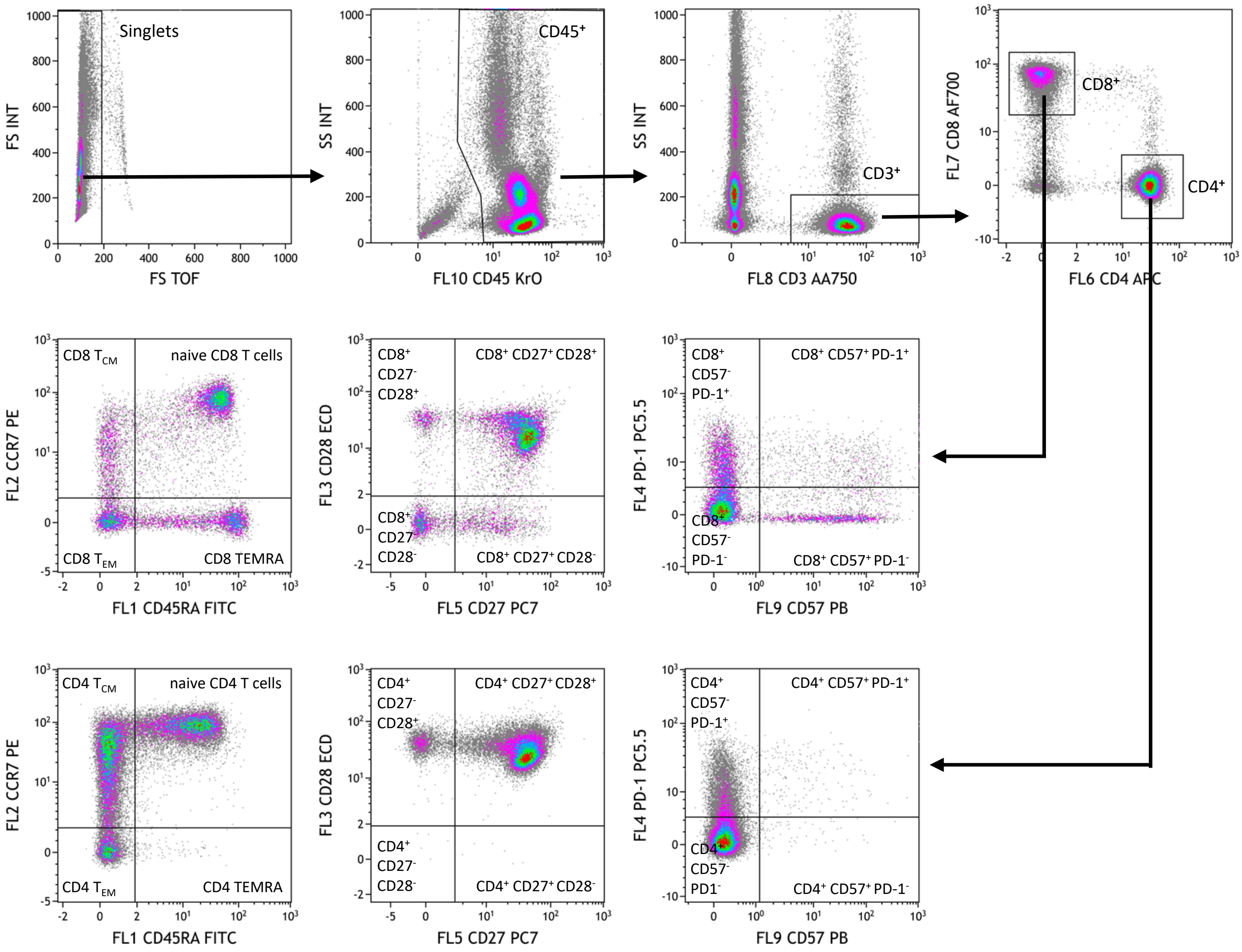

Supplementary Figure 2. *Gating strategy of the DURAClone IM T Cell Subsets Tube.* Cell concentrates were stained using the dried-down DURAClone IM T Cell Subsets Tube (Beckman Coulter) and analyzed by flow cytometry. Sequential gating included selection of singlets, identification of $CD45^+$ leukocytes, and gating of $CD3^+$ T cells. $CD4^+$ and $CD8^+$ T cell subsets were defined within the $CD3^+$ population. Memory T cell subsets were delineated based on CD45RA and CCR7 expression, and CD27/CD28 as well as CD57/PD-1 subpopulations were analyzed within $CD4^+$ and $CD8^+$ T cells. Boolean gates were defined as follows: $CD27^+$ = ($CD27^+$ $CD28^-$) AND ($CD27^+$ $CD28^+$); $CD28^+$ = ($CD27^-$ $CD28^+$) AND ($CD27^+$ $CD28^+$); $CD57^+$ = ($CD57^+$ PD-1^-) AND ($CD57^+$ PD-1^+); PD-1^+ = ($CD57^-$ PD-1^+) AND ($CD57^+$ PD-1^+). A representative gating from one sample is shown.

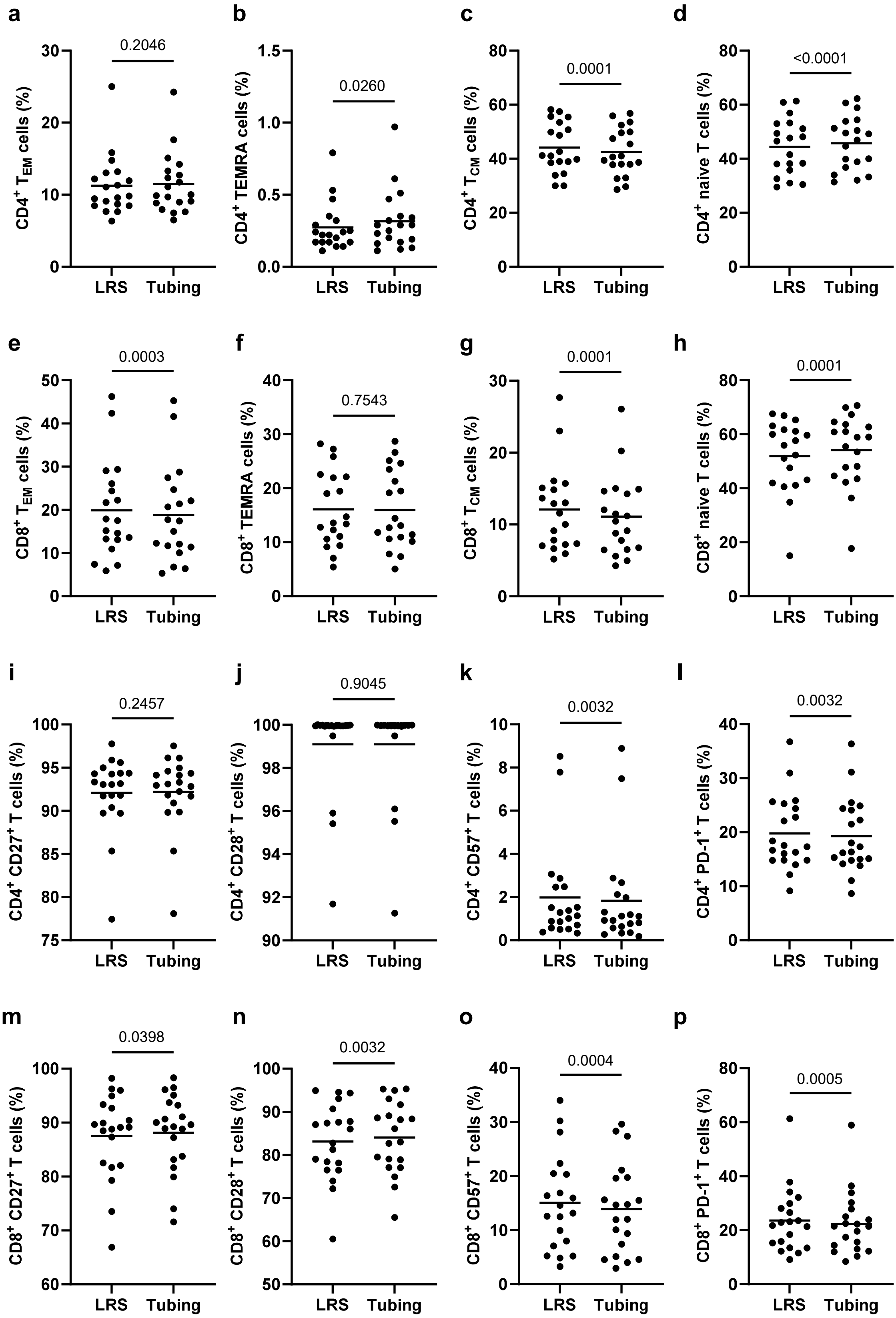

Supplementary Figure 3. *Flow cytometry analysis of CD4⁺ and CD8⁺ T cells revealed no biologically relevant differences in cell concentrates from LRS chambers and tubing sets.* Cell concentrates from matched LRS chambers and tubing sets were stained for flow cytometry analysis using the DURAClone IM T Cell Subsets Tube. Frequencies of CD4⁺ and CD8⁺ T cell sub-populations were determined by manual gating, including **(a)** CD4⁺ T_{EM} cells, **(b)** CD4⁺ TEMRA cells, **(c)** CD4⁺ T_{CM} cells, **(d)** CD4⁺ naive T cells, **(e)** CD8⁺ T_{EM} cells, **(f)** CD8⁺ TEMRA cells, **(g)** CD8⁺ T_{CM} cells, **(h)** CD8⁺ naive T cells, **(i)** CD4⁺ CD27⁺ T cells, **(j)** CD4⁺ CD28⁺ T cells, **(k)** CD4⁺ CD57+ T cells, **(l)** CD4⁺ PD-1⁺ T cells, **(m)** CD8⁺ CD27⁺ T cells, **(n)** CD8⁺ CD28⁺ T cells, **(o)** CD8⁺ CD57⁺ T cells, **(p)** CD8⁺ PD-1⁺ T cells (n=19 for a-h; n = 20 for i-p; paired t-test with Benjamini-Hochberg correction).

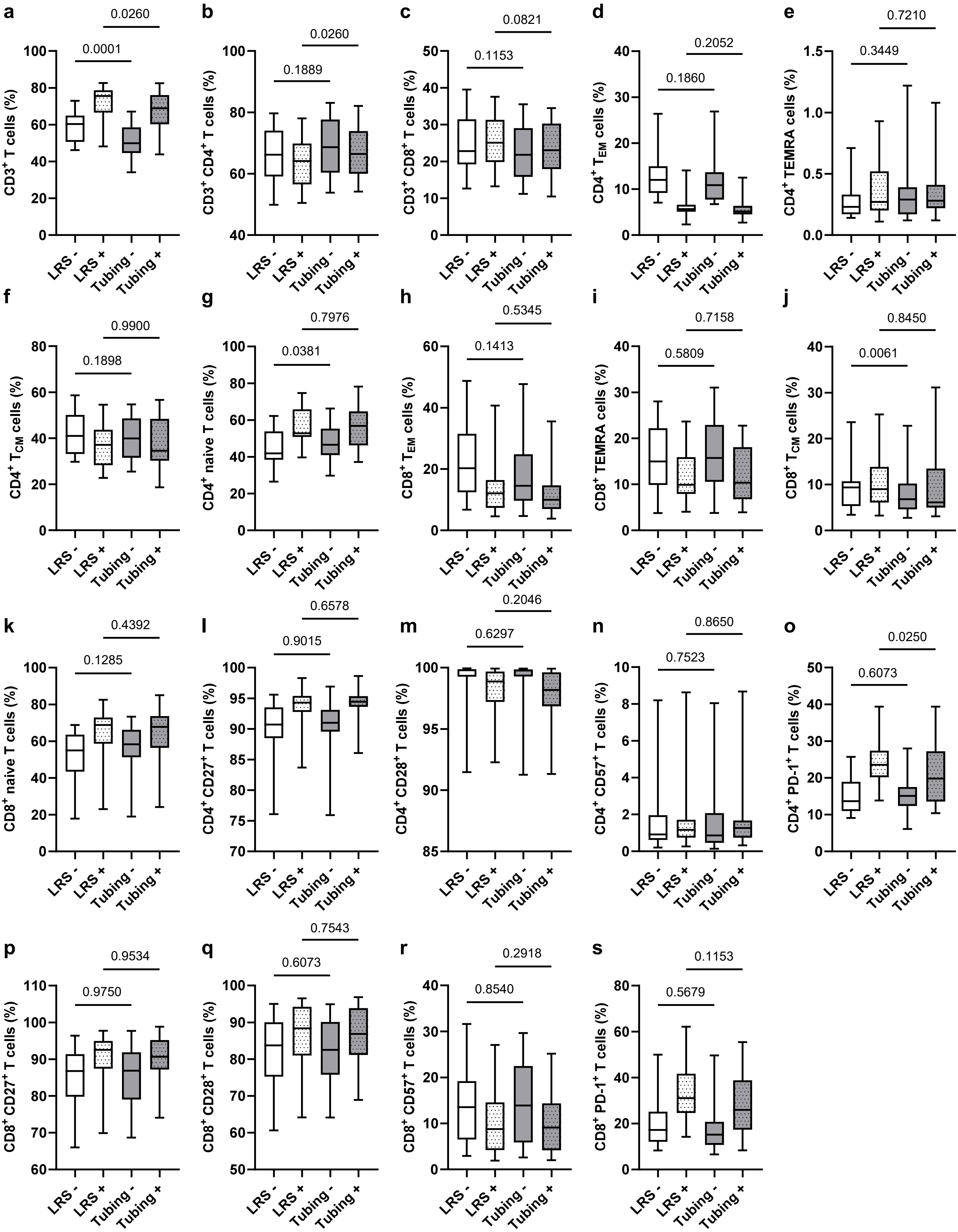

Supplementary Figure 4. *Flow cytometric analysis of cultured PBMCs revealed no differences in responsiveness to PHA-stimulation.* PBMCs from paired LRS chambers and tubing sets were cultured for 22 h in the absence (-) or presence (+) of PHA and stained for flow cytometry using the DURAClone IM T Cell Subsets Tube. Frequencies of T cell populations were determined by manual gating, including **(a)** $CD3^+$ T cells, **(b)** $CD4^+$ T cells, **(c)** $CD8^+$ T cells, **(d)** $CD4^+$ T_{EM} cells, **(e)** $CD4^+$ TEMRA cells, **(f)** $CD4^+$ T_{CM} cells, **(g)** $CD4^+$ naive T cells, **(h)** $CD8^+$ T_{EM} cells, **(i)** $CD8^+$ TEMRA cells, **(j)** $CD8^+$ T_{CM} cells, **(k)** $CD8^+$ naive T cells, **(l)** $CD4^+$ $CD27^+$ T cells, **(m)** $CD4^+$ $CD28^+$ T cells, **(n)** $CD4^+$ $CD57^+$ T cells, **(o)** $CD4^+$ PD-1^+ T cells, **(p)** $CD8^+$ $CD27^+$ T cells, **(q)** $CD8^+$ $CD28^+$ T cells, **(r)** $CD8^+$ $CD57^+$ T cells, **(s)** $CD8^+$ PD-1^+ T cells (n=19 for d-k; n = 20 for a-c and l-s; paired t-test with Benjamini-Hochberg correction).

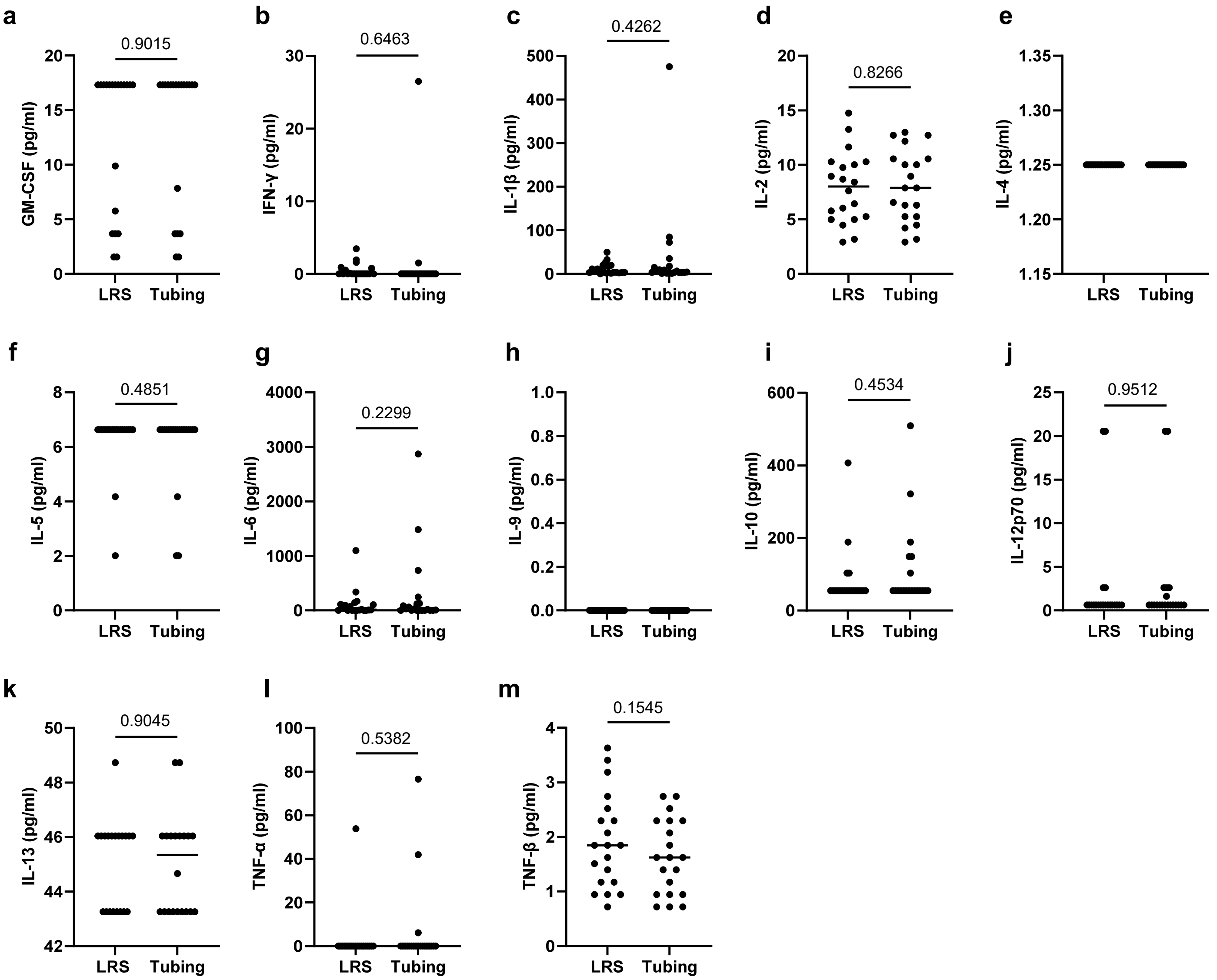

Supplementary Figure 5. *Analysis of cytokine release by non-stimulated control PBMC from LRS chambers and tubing sets.* Untreated control PBMC from paired LRS chambers and tubing sets were cultured for 22 h and cytokine secretion profile was analyzed by Luminex Assay: **(a)** GM-CSF, **(b)** IFN-γ, **(c)** IL-1β, **(d)** IL-2, **(e)** IL-4, **(f)** IL-5, **(g)** IL-6, **(h)** IL-9, **(i)** IL-10, **(j)** IL-12p70, **(k)** IL-13, **(l)** TNF-α, **(m)** TNF-β (n = 20 donors; paired t-test with Benjamini-Hochberg correction).

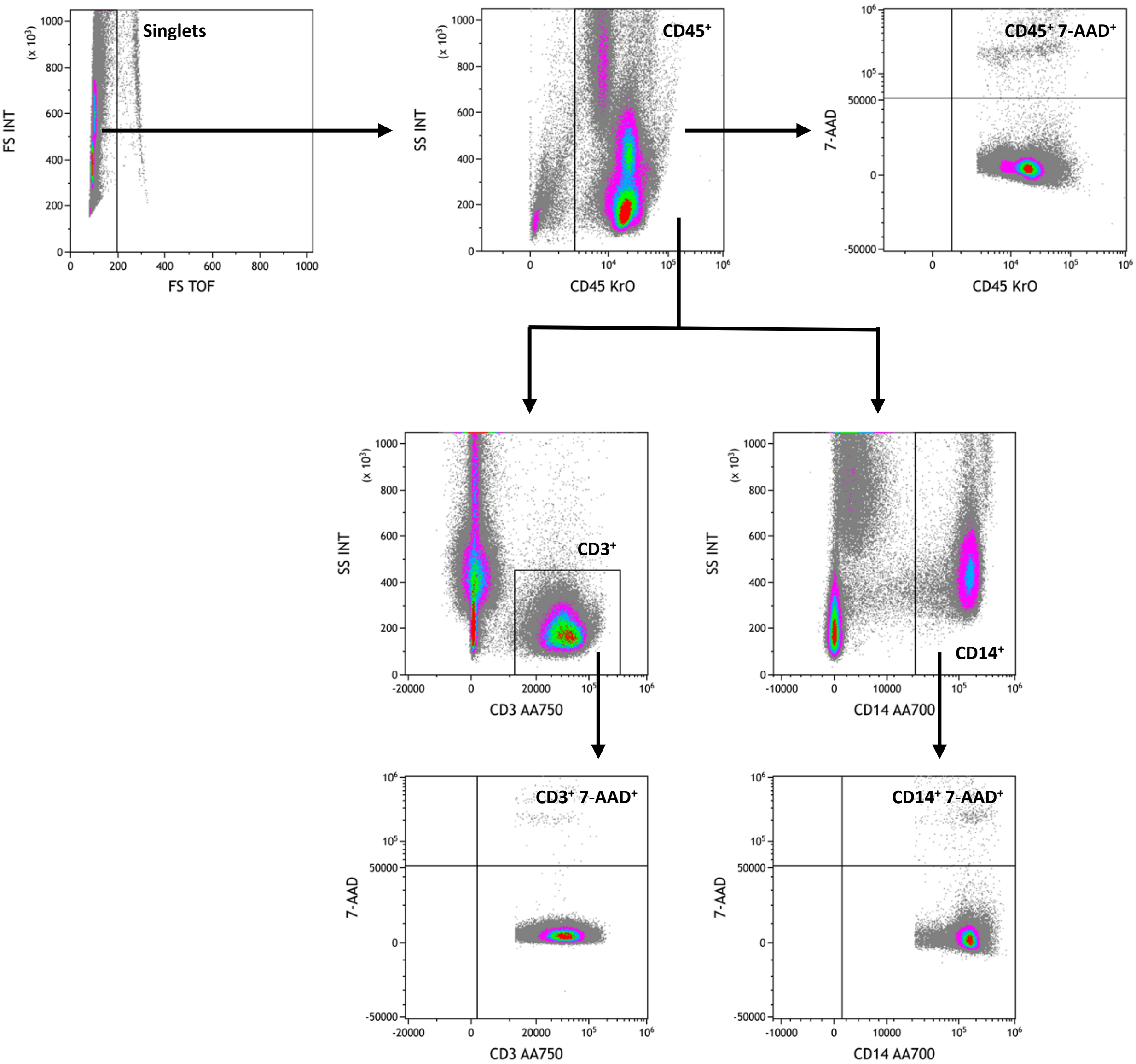

Supplementary Figure 6. *Gating strategy for viability assessment.*
Tubing set samples were stained with antibodies against CD45, CD3, and CD14, together with 7-AAD viability dye. Sequential gating was performed to identify singlets, followed by selection of $CD45^+$ leukocytes. $CD3^+$ T cells and $CD14^+$ monocytes were subsequently gated within the $CD45^+$ population. Viable and non-viable cells were determined by 7-AAD staining, and 7-AAD^+ populations were quantified within the $CD45^+$, $CD3^+$, and $CD14^+$ cell compartments. A representative gating strategy from a single sample is shown.

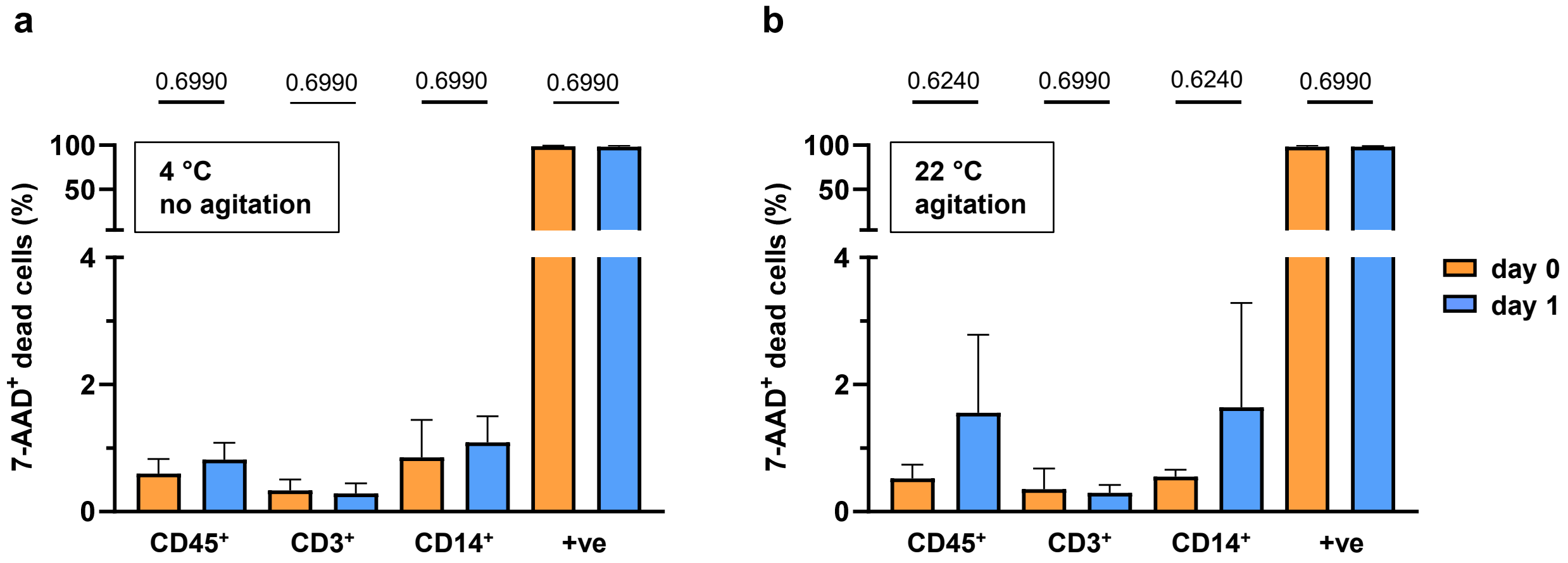

Supplementary Figure 7. *Viability of tubing set cell concentrates after 24 h of storage.* Paired tubing set samples were collected immediately after donation (day 0, orange bars) and after 24 h of storage (day 1, blue bars). Plasma bags containing tubing set cell suspensions were stored either **(a)** at 4 °C without agitation or **(b)** at 22 °C under continuous agitation. Cell viability was assessed by flow cytometry using 7-AAD staining and is shown as the percentage of 7-AAD-positive leukocytes ($CD45^+$), T cells ($CD3^+$), and monocytes ($CD14^+$). Heat-inactivated cells served as positive controls (+ve) (n = 6 donors for each storage condition; paired t-test with Benjamini-Hochberg correction).

Basic donor characteristics

**Donors**	20			
**Gender**				
Female	7 (35 %)			
Male	13 (65 %)			
**Age (years)**	30 (23-61)			
Female	30 (25-61)			
Male	30 (23-49)			
**Donation frequency**	1-5 rounds	6-10 rounds	11-15 rounds	> 15 rounds
(last 12 months)	4 (20 %)	8 (40 %)	6 (30 %)	2 (10 %)
Female	1 (14 %)	4 (57 %)	2 (29 %)	-
Male	3 (23 %)	4 (31 %)	4 (31 %)	2 (15 %)

Supplementary Table 1. *Basic donor cohort characteristics.* Paired LRS and tubing set samples were collected from 20 individual donors. Donor characteristics are presented descriptively. Age is reported as median (minimum - maximum). Donation frequency was calculated based on donations within the 12 months preceding sample collection.

Donor characteristics viability cohort

**Donors**	12			
**Gender**				
Female	3 (25 %)			
Male	9 (75 %)			
**Age (years)**	31 (20-57)			
Female	30 (21-48)			
Male	32 (20-57)			
**Donation frequency**	1-5 rounds	6-10 rounds	11-15 rounds	> 15 rounds
(last 12 months)	2 (17 %)	5 (42 %)	2 (17 %)	3 (25 %)
Female	1 (33 %)	1 (33 %)	1 (33 %)	-
Male	1 (11 %)	4 (44 %)	1 (11 %)	3 (33 %)

Supplementary Table 2. *Donor cohort characteristics for viability assessment.* Paired tubing set samples were collected from 12 individual donors. Donor characteristics are presented descriptively. Age is reported as median (minimum - maximum). Donation frequency was calculated based on donations within the 12 months preceding sample collection.

	Kelly n=31				Kronenberg n=20											
	LRS		Disposable		LRS		Disposable		LRS				Disposable			
	Mean X1	SD	Mean X2	SD	Mean X3	SD	Mean X4	SD	X1-X3	sp	t	p	X2-X4	sp	t	p
Volume (ml)	8,1	0,9	56,4	4,7	8,1	1,0	23,0	14,1	0,0	4,653	0,000	1,000	33,4	47,117	2,472	0,017
WBC ($\times10^9$)	1,3	0,4	1,0	0,6	1,2	0,6	0,7	0,4	0,1	2,361	0,108	0,915	0,3	2,575	0,360	0,721
LYMPH ($\times10^8$)	8,8	3,1	6,2	4,3	8,8	4,2	4,7	2,3	0,0	17,568	0,000	1,000	1,5	18,142	0,283	0,778
NEUT ($\times10^8$)	0,3	0,2	1,4	0,9	0,3	0,2	0,5	0,3	0,0	0,950	-0,048	0,962	0,9	3,587	0,826	0,413
EO ($\times10^6$)	3,1	3,9	10,9	14,2	2,5	1,7	3,6	2,8	0,6	15,953	0,120	0,905	7,3	55,657	0,457	0,650
MONO ($\times10^8$)	3,3	1,7	2,2	1,7	3,1	1,8	1,9	1,1	0,2	8,664	0,095	0,924	0,3	7,444	0,118	0,907

Supplementary Table 3. *Comparison of recovered sample volume and total cell counts with previously reported data.* Recovered volume and absolute numbers of major leukocyte subsets from LRS chamber and tubing set samples were compared with data reported by Kelly et al. [9]. Comparisons were performed using a t-test for recovered volume, total leukocytes (WBC), lymphocytes (LYMPH), neutrophils (NEUT), eosinophils (EO), and monocytes (MONO). Reported statistics include the mean (X), standard deviation (SD), pooled standard deviation (sp), t-score (t), and p-value (p).
